# Genomic epidemiology of third-generation cephalosporin-resistant *Escherichia coli* from companion animals and human infections in Europe

**DOI:** 10.1016/j.onehlt.2025.100971

**Published:** 2025-01-09

**Authors:** Adrien Biguenet, Benoit Valot, Farid El Garch, Xavier Bertrand, Didier Hocquet, the ComPath Study Group

**Affiliations:** a Université de Franche-Comté, UMR-CNRS 6249 Chrono-Environnement, F-25000 Besançon, France; b CHU de Besançon, Hygiène Hospitalière, F-25000 Besançon, France; c Université de Franche-Comté, UFR Santé, CHU de Besançon, Bioinformatique et Big Data Au Service de La Santé, F-25000 Besançon, France; d ComPath Study Group, Bruxelles, Belgium; e Vétoquinol SA, Scientific Division, Lure, France

**Keywords:** Companion animal, ESBL, *Escherichia coli*, Human, Infection, WGS

## Abstract

In high-income countries, dogs and cats are often considered members of the family. Because of this proximity, it has been suggested that pets and humans might exchange bacterial species from their gut microbiota, with multidrug resistant bacteria being of particular concern. The aim of this study was to compare the genomes of third-generation cephalosporin-resistant (3GC-R) *Escherichia coli* responsible for human and pet infections in Europe.

Whole-genome sequencing data from 3GC-R *E. coli* isolated from clinical samples of humans, dogs and cats, and published in eight European studies were re-analyzed using bioinformatics tools. The acquired genes responsible for 3GC-R were identified. The sequence type (ST) of all genomes were assessed by multilocus sequence typing. Alpha and beta diversities were measured within and between the two populations.

We included genomes of 1327 3GC-R *E. coli* isolated from humans and animals with 109 (8.2 %) being responsible for infections in dogs and cat, and 1218 (91.8 %) responsible for human infections. Alpha diversity analysis suggested greater diversity within ST and 3GC-R genes in the animal population. Beta diversity analysis by principal coordinate analysis separated animal and human strains. ST131 was more abundant in human strains (43.4 %) than in animal strains (14.7 %) (*p* < 0.001). Six STs, including ST372, were identified almost exclusively in 3GC-R *E. coli* from animal origin. The *bla*_CTX-M-15_ gene was more frequent in humans (49.24 %) than in companion animals (17.9 %) (*p* < 0.001). The resistance genes *bla*_CMY-2_ (30.8 %) and *bla*_CTX-M-1_ (15.4 %) were more frequent in *E. coli* isolated from pets (p < 0.001).

We found that populations of 3GC-R *E. coli* responsible for human and pet infections in Europe do not overlap. Although it cannot rule out occasional transmission of bacteria between pets and humans within a household, it suggests that dogs and cats are not a major source of human infection with this antibiotic-resistant pathogen.

## Highlights

• Populations of 3GC-R *E. coli* responsible for human and pet infections do not overlap.
• The *bla*_CTX-M-15_ gene was more common in *E. coli* from humans than from pets.
• The *bla*_CMY-2_ and *bla*_CTX-M-1_ resistance genes were more common in *E. coli* from pets than from humans.
• ST131 *E. coli* were isolated from human clinical samples more often than clinical samples from pets.

## 1 Introduction

Extended-spectrum-β-lactamase (ESBL) and plasmid-mediated cephalosporinase (pAmpC)-producing *Escherichia coli* have been reported worldwide in humans, companion animals, livestock, food, and the environment [1]. These bacteria are considered a major threat to human health by the World Health Organization (WHO) with a variable distribution between regions and hosts [2,3]. In high-income countries, dogs and cats can be considered family members. With a European population of around 746 million people, the European Pet Food Industry (https://europeanpetfood.org/about/statistics) reports 104 million dogs and 127 million cats across the continent. The close proximity between pets and humans has led to suggestions that these companions and humans may share similar bacteria [4]. Transmission events have been observed between pets and their owners [[5], [6], [7]]. Although such transmission has also been occasionally documented between livestock and farmers [8,9], the extent to which livestock is a source of human infection remains controversial [10]. Hence, third-generation cephalosporin-resistant (3GC-R) *E. coli* isolates from livestock and human mostly belong to separated populations in Europe [1,10,11]. *E. coli* populations differ from one host to another. *E. coli* producing pAmpC is more frequently responsible for infections in companion animals than in humans [1]. Conversely, ST131 producing CTXM-15 is the main 3GC-R *E. coli* clone responsible for infection in humans [12], but not in companion animal [1,13,14].

Whole genome sequencing (WGS) analyses of 3GC-R *E. coli* from diseased dogs and cats without sample bias are rare [15,16] and mainly concern fecal carriage from healthy or diseased animals [5,6,17]. Here, we compared the populations of 3GC-R *Escherichia coli* responsible for human and pet infections in Europe, by re-analysing 1327 bacterial genomes from a European collection of this antibiotic-resistant pathogen.

## 2 Materials and methods

### 2.1 Genome inclusion criteria

To compare *E. coli* strains responsible for infections in animals and humans, we conducted a comprehensive search of the PubMed database covering the period from 2013 to 2023. Our search criteria were “(*Escherichia coli* OR Enterobacteriaceae OR Enterobacterales) AND (multidrug OR cephalosporin OR AmpC OR ESBL) AND (genome OR WGS OR Illumina).” This search yielded 4295 studies. We specifically searched for studies that included unbiased collections of 3GC-R *E. coli* with publicly available complete genomes and isolated from infections in humans, dogs, and cats within the European region. Title, abstract, and full paper screening excluded 4259, 16, and 8 studies, respectively (Fig. 1 in the Supplementary Data). Four studies were excluded because WGS data were not available. Ultimately, we identified and included eight studies that met our criteria.

One hundred and thirteen genomes of *E. coli* responsible for dog and cat infections in twelve countries were selected from two publications [15,16], and the ComPath III study [18] (Supplementary Data). Additionally, 1822 genomes of *E. coli* responsible for human infections in seven countries were selected from six publications [14,[19], [20], [21], [22], [23]]. To ensure temporal congruence, human-origin strains were restricted to those sampled between 2008 and 2018 to match the isolation dates of animal-origin *E. coli* isolates (Table 1, Table 2, and Table 3 in the Supplementary Data).

**Table 1:** Distribution and comparison of *Escherichia coli* ST131, *bla*_CTX-M-1_, *bla*_CTX-M-15_ and *bla*_CMY-2_ between the Northern and Southern regions for human and animal clinical samples. Chi2 test with *p* < 0.05 was considered significant.

	Host (Number of *E. coli* genomes)	ST131 n (%)	*bla*_CTX-M-1_ n (%)	*bla*_CTX-M-15_ n (%)	*bla*_CMY-2_ n (%)
Northern region	Humans (719)	366 (50.07)	23 (3.15)	429 (58.69)	34 (4.65)
	Pets (43)	5 (11.63)	6 (13.95)	8 (18.60)	9 (20.93)
	Pets *vs.* humans	p < 0.001	*p* = 0.004	*p* < 0.001	< 0.001
Southern region	Humans (348)	95 (27.30)	41 (11.78)	123 (35.34)	34 (9.77)
	Pets (65)	9 (13.85)	11 (16.92)	9 (13.85)	22 (33.85)
	Pets *vs.* humans	*p* = 0.022	p = 0.251	< 0.001	< 0.001

To limit the impact of variations in antimicrobial resistance between European countries, we grouped *E. coli* genomes into three distinct regions: Northern Europe (Belgium, Denmark, Germany, the Netherlands, Norway and the United Kingdom), Eastern Europe (Czech Republic, Estonia, Hungary, Latvia, Lithuania and Poland), and Southern Europe (France, Italy, Spain and Switzerland). Meta-data for all strains including the site of infection, and information on the studies used are available in the Supplementary Data.

### 2.2 Genome sequencing, assembly, quality assessment, and identification of duplicate isolates

Pair-ends short read WGS (5 collections) or assembly data (2 collections) were downloaded from the NCBI or ENA databases. ComPath III WGS pair-ends short reads were generated for this study. More information on sequencing and assembly can be found in the Supplementary Table 5. Available pair-ends short reads were subsampled to normalize coverage to 100×, trimmed using Trimmomatic v0.39 [24], and then assembled using SPAdes v3.13.1 [25]. Contigs with coverage <2 and length < 300 bp were filtered out. The quality of the assemblies was assessed using Quast v5.2.0 [26]. Assembled genomes with >900 contigs or a genome size ≥6,000,000 bp were excluded from further analysis. The calculation of the cgMLST matrix distance was performed by pyMLST v2.1.5 [27], using the *E. coli* core genome multilocus sequence typing (cgMLST) scheme available at https://www.cgmlst.org/. We searched potential cross-event transmission within each collection, where two isolates were considered as duplicate if they were isolated within <2 years in the same country and if they had ≤10 alleles identified by cgMLST [28]. Only the oldest strains of each event were retained.

### 2.3 Bioinformatics analysis

Phylogroup, *fimH* typing and MultiLocus Sequence Typing (MLST, using Achtman scheme) were determined using ClermonTyping v3 [29], *fimH* typer [30] and pyMLST v2.1.5 [27], respectively. Antibiotic resistance genes or point mutations were identified with AMRFinderPlus v3.11.14 and database version 2023-07-13.2 [31] with a minimum identity percentage of 90 % and a minimum coverage length of 60 %. Resistance genes identified with less than 100 % identity were manually searched in the NCBI database using blastp.

Phylogenetic relationships among the ST131 *E. coli* strains were inferred using MrBayes software (version 3.2.7) [32]. First, multiple sequence alignment files were generated from the whole genome sequences. Genes present in at least 95 % of the strains and having less than 2 mutations per 100 base pairs were selected using pyMLST [27]. Phylogenetic analysis was performed using the HKY85 + G model. Two independent Markov chain Monte Carlo chains were run simultaneously for 1000,000 generations, with samples every 1000 generations. Convergence of the chains was assessed using the minimum effective sample size (ESS ≥ 100) and the potential scale reduction factor (PSRF close to 1). The first 25 % of samples were discarded as burn-in, and a consensus tree was constructed from the remaining samples using MrBayes. The tree was rooted using a ST73 *E. coli* strain from our study (BioSample SAMEA104060572) and visualized and annotated using iTol v6.6 [33].

### 2.4 Diversity analysis between animal and human *E. coli*

Alpha diversity metrics, including species richness (SR), the exponential of Shannon entropy (SE), and the inverse of Simpson diversity (SD), were computed as per established methods [16,34,35] using the R package vegan v2.5–686 [36]. The size of the *E. coli* population of human origin was sub-sampled to that of the *E. coli* population of animal origin. Sub-sampling was done with 10,000 permutations without replacement. Diversity was considered significantly different between the animal and human sectors if the measured diversity of animal *E. coli* population did not fall within the 95 % confidence intervals of the measured diversity of human *E. coli* population [35]. Confidence intervals were calculated using bootstrap with 1000 iterations with boot R package. Beta diversity was calculated using a matrix of counts for host and region parameters, with sequence types and 3GC-R genes as ‘species’. The matrices were transformed using Hellinger's method prior to Principal Coordinate Analysis (PCoA) using the R vegan package.

### 2.5 Statistical analysis

Fisher's Exact test or Chi2 test were used for categorical variables as appropriate. For all analyses, a *p*-value <0.05 was considered statistically significant. Analyses were performed using R v4.2.3.

## 3 Results

### 3.1 Bacterial isolates

**Fig. 1:**
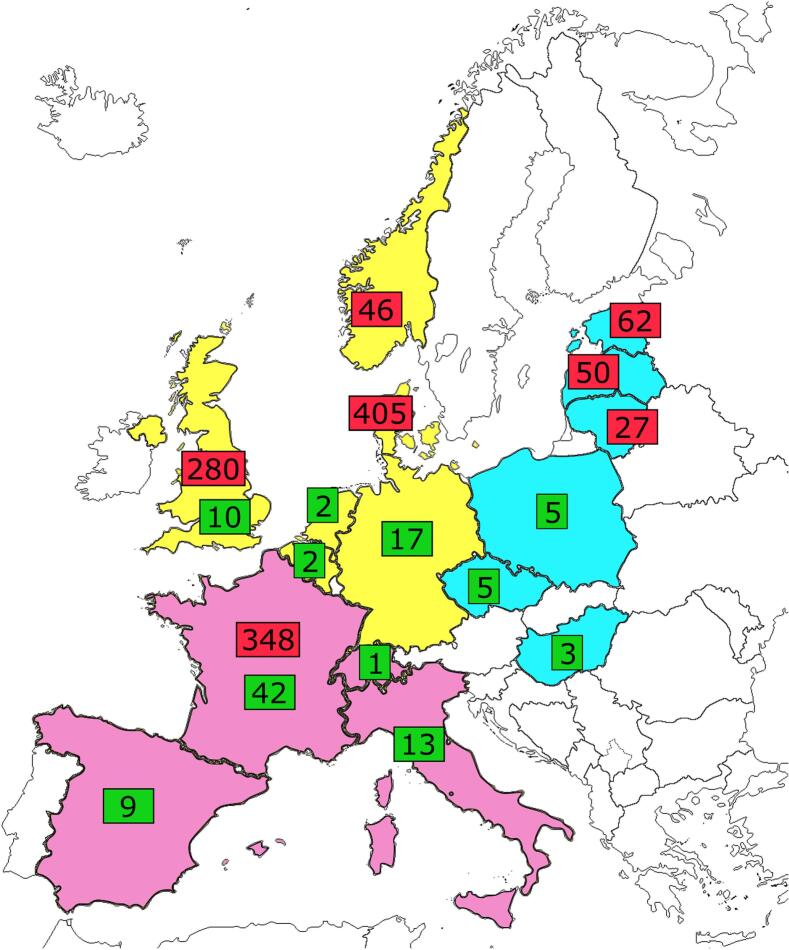
European map of 3GC-R *E. coli* genomes included in the study by country. The European continent is divided into Northern region (yellow), Southern region (pink), and Eastern region (blue). Red and green rectangles give the number of 3GC-R *E. coli* genomes retrieved from human and animal infections, respectively, in each country. (For interpretation of the references to colour in this figure legend, the reader is referred to the web version of this article.)

A total of 1822 genomes were available from the 8 included studies. After bioinformatics analysis, we excluded 257 genomes (14.1 %) that did not harbor 3GC-R genes and 238 duplicated genomes (13.1 %). Of the 1327 genomes included, 1218 (91.8 %) were from human infections and 109 (8.2 %) were from infections in dogs (*n* = 74, 68.0 %) or cats (*n* = 35, 32.1 %). The included genomes were classified according to their geographical origin: 762 (including 31 from animals) were from Northern Europe, 413 (including 65 from animals) from Southern Europe, and 152 (including 13 from animals) from Eastern Europe. Fig. 1 shows the number of genomes included per country.

### 3.2 Population structure of 3GC-R *E. coli*

The three most common phylogroups identified in *E. coli* from human origin were phylogroup B2 (*n* = 651, 53.4 %), followed by phylogroup D (*n* = 203, 16.7 %) and phylogroup A (*n* = 129, 10.6 %). In animal infections, the *E. coli* population was dominated by phylogroup B2 (*n* = 35, 32.1 %), followed by phylogroup B1 (*n* = 21, 19.3 %) and phylogroup D (n = 21, 19.3 %). While phylogroup B2 *E. coli* dominated human and animal isolates, B2 *E. coli* prevalence was significantly higher in isolates of human origin (*p* < 0.001). Phylogroup B1 and F were more frequent in the animal population than in the human population (p < 0.001 and *p* = 0.023, respectively). Other phylogroups were equally distributed between the companion animal and human populations.

**Fig. 2:**
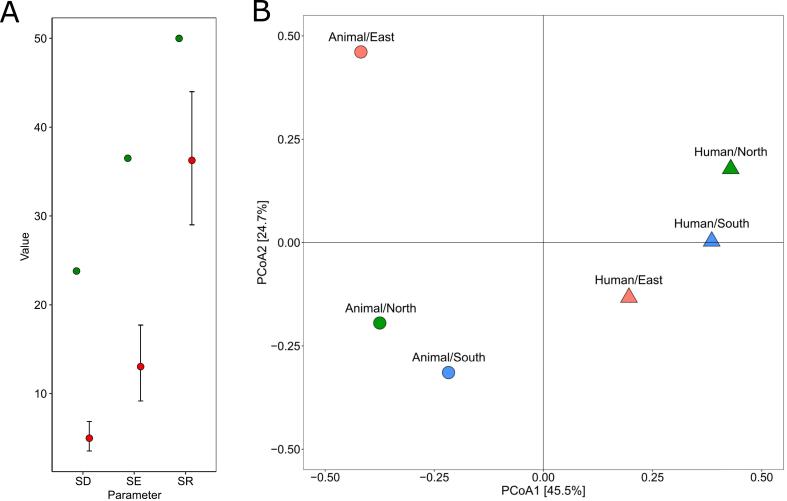
Comparison of the diversity of the sequence type of *E. coli* genome population from human (*n* = 1218) and animal (*n* = 109) infections. (A) *E. coli* alpha diversity of animal origin (green) and human origin (red). (B) Beta diversity by principal coordinate analysis using a Hellinger-transformed distance matrix. SD: inverse of Simpson diversity; SE: exponential of Shannon entropy; SR: Species richness. (For interpretation of the references to colour in this figure legend, the reader is referred to the web version of this article.)

Alpha diversity analysis of ST (Fig. 2) demonstrated significantly higher diversity in 3GC-R *E. coli* isolated from animal infections (SR = 50, SD = 23.8, and SE = 36.5) compared to *E. coli* isolated from human infections (SR = 36.3, SD = 4.98, and SE = 13.03). Beta diversity analysis depicted in Fig. 2 revealed a separation between *E. coli* genomes derived from animals and humans along the first axis.

The population of 3GC-R *E. coli* isolated from human infections was dominated by ST131 (*n* = 529, 43.4 %) followed by ST38 (*n* = 79, 6.5 %), ST405 (*n* = 41, 3.4 %), ST69 (*n* = 40, 3.3 %), and ST648 (*n* = 35, 2.9 %). The population of 3GC-R *E. coli* from animals was mainly composed of ST131 (*n* = 16, 14.7 %), followed by ST372 (*n* = 6, 5.5 %) and ST648, ST88, ST744, ST224, ST1431 (n = 4, 3.7 % each). ST131 isolates were more prevalent in the human 3GC-R *E. coli* population (*p* < 0.001), while some STs were more frequently associated with companion animal infections (ST372, ST1431, ST224, ST973, ST1011, and ST1485; p < 0.001). Fig. 4 showed that ST131 *E. coli* of animal origin were overrepresented in *fimH*22 (18.2 %; *n* = 22) compared to *fimH*41 (3.6 %; *n* = 56; *p* = 0.049) and *fimH*30 (2.1 %; *n* = 467; *p* < 0.01).

### 3.3 Genes conferring resistance to third-generation cephalosporins

**Fig. 3:**
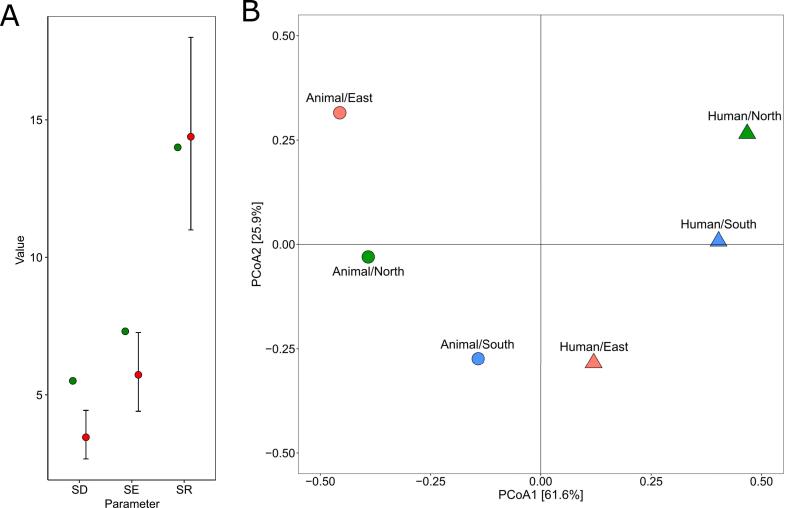
Comparison of the diversity of 3GC-R genes from human (n = 1218) and animal (n = 109) *E. coli*. (A) *E. coli* alpha diversity of animal origin (green) and human origin (red). (B) Beta diversity by principal coordinate analysis using a Hellinger-transformed distance matrix. SD: inverse of Simpson diversity; SE: exponential of Shannon entropy; SR: Species richness. (For interpretation of the references to colour in this figure legend, the reader is referred to the web version of this article.)

Alpha diversity of genes conferring resistance to 3GC was higher in *E. coli* of animal origin (SR = 14.00, SD = 5.51 and SE = 7.31) than in those of human origin (SR = 14.39, SD = 3.46, and SE = 5.73) (Fig. 3). The beta diversity analysis shown in Fig. 3 also revealed a separation between isolates recovered from animals and humans according to the first axis.

**Fig. 4:**
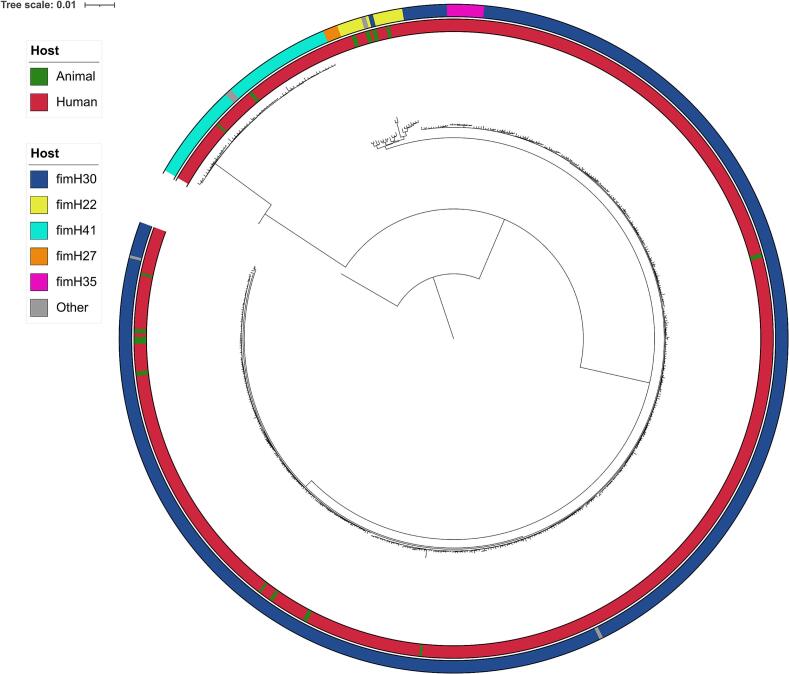
Phylogenetic tree of 545 *E. coli* ST131 strains included in this study. The tree was generated with a Bayesian approach using the HKY85 + G model and rooted with the genome of a ST73 *E. coli* strain (BioSample SAMEA104060572). In the inner circle, genomes of isolates retrieved from animals and human are indicated in green and red, respectively. (For interpretation of the references to colour in this figure legend, the reader is referred to the web version of this article.)

Of the 1249 genes conferring resistance to 3GC identified in human-derived *E. coli*, 90.7 % encoded an ESBL (*n* = 1133) and 9.29 % encoded a pAmpC (*n* = 116). The most prevalent genes encoding resistance to 3GC in human-derived *E. coli* were *bla*_CTX-M-15_ (*n* = 615; 49.2 %), *bla*_CTX-M-14_ (*n* = 185; 14.8 %), *bla*_CTX-M-27_ (*n* = 102; 8.2 %), *bla*_CMY-2_ (*n* = 87; 7.0 %), *bla*_CTX-M-1_ (*n* = 74; 5.9 %). In companion animals, 117 genes associated to 3GC-R genes were identified, including ESBLs (*n* = 81; 69.2 %) and pAmpC (*n* = 36; 30.8 %). The most frequent genes responsible for 3GC-R in *E. coli* from companion animals were *bla*_CMY-2_ (n = 36; 30.8 %), *bla*_CTX-M-15_ (*n* = 21; 17.9 %), *bla*_CTX-M-14_ (n = 18; 15.4 %), *bla*_CTX-M-1_ (n = 18; 15.4 %), *bla*_CTX-M-27_ (n = 8; 6.8 %). The resistance genes *bla*_CMY-2_ (*p* < 0.001) and *bla*_CTX-M-1_ (p < 0.001) were significantly more prevalent in *E. coli* from companion animals than from humans, while *bla*_CTX-M-15_ was significantly more frequent in human infections (p < 0.001). *bla*_CTX-M-27_ and *bla*_CTX-M-14_ were equally distributed in the two collections (*p* = 0.743 and 0.976, respectively).

### 3.4 Comparison of *E. coli* genomes from human and animal infections by region of isolation

The distribution of ST131, *bla*_CTX-M-1_, *bla*_CTX-M-15_, and *bla*_CMY-2_ differed between regions (p < 0.001). To test whether the observed differences between genomes of *E. coli* of human and animal origin were region-specific, we compared the distribution of STs and genes encoding resistance to 3GC between the Northern and Southern regions (Table 1). The results within regions were consistent with those observed in the mixed genome analysis encompassing all of Europe, with the exception of *bla*_CTX-M-1_ within the Southern region (*p* = 0.251). The limited number of *E. coli* genomes of animal origin from Eastern region (*n* = 13) impeded comparisons with this area.

### 3.5 Genomic comparison of *E. coli* isolates from urinary tract infections

Of the 173 isolates (13.0 %) responsible for urinary tract infections, 38 (22.0 %) came from veterinary clinical samples and 135 (78.0 %) came from human clinical samples. Compared genomes isolated within the Northern (8 from companion animals and 80 from humans) and Southern regions (23 from companion animals and 55 from humans), we found that *bla*_CTX-M-1_ (*n* = 4, 12.9 % *vs.* n = 4, 2.96 %) and *bla*_CMY-2_ (*n* = 16, 51.6 % *vs. n* = 5, 3.7 %) were more abundant in companion animal isolates (*p* = 0.041 and *p* < 0. 001, respectively). Conversely, *bla*_CTX-M-15_ (n = 4, 12.9 % *vs. n* = 96, 71.1 %) and ST131 (*n* = 3, 9.7 % *vs. n* = 87; 64.4 %) were significantly more frequent in human isolates (*p* < 0.001 and p < 0.001, respectively).

## 4 Discussion

In this comparative analysis, we explored the population structure of 3GC-R *E. coli* isolated from European clinical samples of humans and pets. *E. coli* isolates responsible for infections in dogs and cats differed in both ST and 3GC-R encoding genes when compared to strains isolated from human clinical samples. The alpha diversity of 3GC-R *E. coli* isolated from animals was higher than that of those isolated from humans, indicating a more diverse and equitable population with a lower dominance of specific STs and/or 3GC-R genes in *E. coli* of animal-origin. One hypothesis would be that the rare interactions between domestic animals in Europe favor the diversity of 3GC-R *E. coli* of animal origin, whereas frequent human-to-human interactions may facilitate the spread of a dominant clone across the continent.

The human-related 3GC-R *E. coli* population mostly harbors *bla*_CTX-M-15_, while the pet-related *E. coli* population mostly harbors *bla*_CMY-2_ and *bla*_CTX-M-1_ as in the livestock population (*bla*_CMY-2_ in poultry, and *bla*_CTX-M-1_ in cattle and poultry) [2]. Genome-based studies suggest that the human and livestock populations have distinct populations with rare cross-transmission events [10,11]. Although genes responsible for 3GC-R in *E. coli* from humans and companion animals remain distinct, one might expect that humans would share more resistance genes with companion animals than with livestock, as humans live closely with dogs and cats [11].

We found differences in population structure of the 3GC-R *E. coli* population responsible for pet and human infections. Six STs were more prevalent in companion animals than in humans, including ST372, a major clone responsible for canine infections. In addition, the ST372 lineages found in dogs and humans have been found to be distinct [4,37]. Although ST131 was the predominate ST of *E. coli* isolated from humans and companion animals in our study, this ST was detected more often in isolates of human origin. As for the 3GC-R related genes, the proportion of ST131 in companion animals is between those found in humans and livestock. Hence, *E. coli* ST131 carrying *bla*_CTX-M-15_ represented 69.9 % (370/529) of all ST131 infections of human origin in our study, while we found only two *E. coli* ST131 *bla*_CTX-M-15_ of the 16 ST131 genomes isolated from pet infections (12.5 %) (*p* < 0.001). As *bla*_CTX-M-15_ is frequently chromosomal in *E. coli* ST131 [16,38], it suggests that transmissions of *E. coli* ST131 *bla*_CTX-M-15_ between humans and companion animals are rare events. Hence, the phylogenetic tree of the 545 genomes of *E. coli* ST131 of the present collection showed a higher prevalence of animal genomes in the *fimH*22 cluster, emphasizing the difference between animal- and human-related 3GC-R *E. coli* strains. However, animal-related strains in the *fimH*41 and *fimH*30 clusters support cross-transmission between companion animals and humans.

Our study had several limitations. The small number of genomes of 3GC-R *E. coli* strains isolated from companion animal infections limited the analysis of subgroups. We found no studies conducted in the same country with enough strains to compare genomes. In addition, strains have been isolated from different infection sites while epidemiology may differ between extra- and intra-intestinal infections [39]. To limit these biases, *E. coli* populations from humans and companion animals were compared within the Northern and Southern regions of Europe which confirmed the differences observed globally between human and companion animal populations of *E. coli*. In addition, our genomic comparison of the subset of strains responsible for urinary tract infections retrieved similar results to those with the general population of 3GC-R *E. coli*. We believe that although a macro analysis has limitations, our methodology allows us to consider the two populations as different. In *E. coli,* genes encoding resistance to 3GC are often located on plasmids. The short reads produced by Illumina methods impede the plasmid reconstruction or the association of 3GC-R associated genes with the plasmid replicon. Although we could not assess plasmid transmission between human and companion animal *E. coli* populations, we demonstrated that the strains were genetically distinct, and that the 3GC-R associated genes differed between the two populations. Although cross-transmission events cannot be excluded, our results do not support a large diffusion of a plasmid between the two *E. coli* populations (human *versus* animal origin) investigated in this study.

## 5 Conclusion

With this large European study, our findings suggest that human and companion animal *E. coli* populations responsible for infections differ in ST and genes encoding resistance to 3GC. Although there is no overlap between European populations of 3GC-resistant *E. coli* responsible for infections in humans and companion animals, occasional transmission of clones within households cannot be excluded. However, pets are unlikely to be a significant source of human infections.

## Funding

This work was supported by 10.13039/100014851Bayer Animal Health GmbH, Boehringer Ingelheim Animal Health, Ceva Santé Animale, Elanco Animal Health, Fatro, MSD Animal Health, Vetoquinol, Virbac and Zoetis, all members of CEESA (Executive Animal Health Study Center).

## CRediT authorship contribution statement

**Adrien Biguenet:** Writing – original draft, Formal analysis. **Benoit Valot:** Methodology, Formal analysis. **Farid El Garch:** Writing – review & editing, Funding acquisition. **Xavier Bertrand:** Writing – review & editing, Supervision, Methodology. **Didier Hocquet:** Writing – review & editing, Supervision, Conceptualization.

## Declaration of competing interest

The Compath group reports financial support was provided by Bayer Animal Health GmbH. The Compath group reports financial support was provided by Boehringer Ingelheim Animal Health. The Compath group reports financial support was provided by Ceva Santé Animale. The Compath group reports was provided by Elanco Animal Health. The Compath group reports financial support was provided by Fatro. The Compath group reports financial support was provided by MSD Animal Health. The Compath group reports financial support was provided by Vétoquinol France. The Compath group reports financial support was provided by Virbac. The Compath group reports financial support was provided by Zoetis. Farid El Garch reports a relationship with Vétoquinol France that includes: employment. If there are other authors, they declare that they have no known competing financial interests or personal relationships that could have appeared to influence the work reported in this paper.

## Data Availability

Data will be made available on request.

## Appendix A Supplementary data

Supplementary material 1 Image 1

Supplementary material 2 Image 2
