# Supplementary material for: Genomic epidemiology of third-generation cephalosporin-resistant Escherichia coli from companion animals and human infections in Europe
Source: One Health. 2025 Jan 9;20:100971. doi: 10.1016/j.onehlt.2025.100971 (PMC11787529; doi:10.1016/j.onehlt.2025.100971)
Supplement: Supplementary file 1 — Supplementary material 1 [file mmc1.docx]

**SUPPLEMENTARY DATA**

# Compath III material and methods

**Bacterial isolates.** The Executive Animal Health Study Center (CEESA) monitors a program (ComPath) to survey antimicrobial susceptibility of bacterial pathogens isolated from dogs and cats suffering from respiratory tract infection, urinary tract infection and skin and soft tissue infection in Europe. Local laboratories in each country were responsible for recovery and identification of bacterial isolates. Samples were collected by veterinary practitioners in their daily practice in 12 European countries: Belgium, Czech Republic, France, Germany, Hungary, Italy, The Netherlands, Poland, Spain, Sweden, Switzerland, and the United Kingdom. Only one animal per household, breeder, or kennel was included and one sample per animal. Chronically-diseased animals were excluded.

**Antimicrobial susceptibility testing.** Bacterial species were confirmed by matrix-assisted laser desorption/ionization time-of-flight mass spectrometry (MALDI-TOF MS) (Microflex; Bruker Daltonics, Bremen, Germany) according to the manufacturer’s recommendations. MICs of cefotaxime (CTX) and ceftazidime (CAZ) were determined by agar dilution and interpreted according to EUCAST (European Committee on Antimicrobial Susceptibility Testing. Breakpoint tables for interpretation of MICs and zone diameters. Version 11.0, January 2021). 3GC-resistant Enterobacterales (*i.e.*, with CTX MIC > 2 mg/L and/or CAZ MIC > 4 mg/L) were selected for genome sequencing.

This study targeted 42 *Escherichia* coli with CTX MIC > 2 mg/L and/or CAZ MIC > 4 mg/L, isolated between 2017 and 2018 in 9 European countries (Belgium, France, Germany, Hungary, Italy, The Netherlands, Poland, Spain, and the United Kingdom).

# Table I. Number and origin of the 1713 bacterial genomes from *E. coli* strains of human origin included in this study.

| **Country** | **Study** | **Number of strains per study** | **Number of strains per country** |
| --- | --- | --- | --- |
| France | Zamudio et al. | 263 | 348 |
|  | de Lastours et al. | 85 |  |
| The United Kingdom | Lipworth et al. | 224 | 280 |
|  | Kallonen et al. | 56 |  |
| Danemark | Roer et al. | 405 | 405 |
| Estonia | Sepp et al. | 62 | 62 |
| Latvia |  | 50 | 50 |
| Lithuania |  | 27 | 27 |
| Norway |  | 46 | 46 |

# Table II. Method used to select *E. coli* isolates of human origin in the included studies

| **Study** | **Method used to select bacterial isolates** | **Years of sampling** |
| --- | --- | --- |
| Zamudio et al. | - *Escherichia coli* strains with ESC-R mechanism were sampled from 11 of the 13 regions of France. - Strains mainly originating from the national surveillance of antibiotic resistance sent by French laboratories. | 2008 – 2016 |
| Lipworth et al. | - Unbiased sampling of *E. coli* without selection according to resistance mechanisms. - All *E. coli* isolates that caused bloodstream infections between 2008 and 2018 from four hospitals and all community health care facilities in the county of Oxfordshire. | 2008 – 2018 |
| Kallonen et al. | - Unbiased sample of *E. coli* from bloodstream infections regardless of their mechanism of resistance to antibiotics. - BSAC *E. coli* strains were from 11 centers across England from the Bacteremia Resistance Surveillance Programme. - ‘CUH’ *E. coli* strains were from the diagnostic laboratory at Cambridge University Hospitals. Every third isolate associated with bacteremia was included. | 2008 – 2012 |
| Roer et al. | - *E. coli* strains from bacteremia were collected from 11 of the 12 departments of clinical microbiology in Denmark. - These strains were selected for resistance to 3GCs. Strains resistant to at least one 3GC were included in the study. Only the first isolate per patient was included. | 2014 – 2015 |
| Sepp et al. | - *E. coli* strains were screened for resistance to 3GCs. - 21 hospitals from 5 countries of Northern/Eastern Europe located in Estonia (n = 5), Latvia (n = 4), Norway (n = 1) and St. Petersburg, Russia (n = 8) were included. Strains from Lithuania were not included due to the low number of strains. - Strains were sampled from outpatients or hospitalized patients of all ages. *E. coli* strains were collected from blood, pus, urine, and respiratory tract. Only clinically relevant samples were included. | 2012 |
| de Lastours  et al. | - *E. coli* strains from bacteriemia were included regardless of their resistance to antibiotics. - Strains were obtained from the SEPTICOLI study conducted in seven teaching hospitals in Paris. | 2016 – 2017 |

**Table III. Number and origin of the 109 bacterial genomes from *E. coli* strains of animal origin included in this study.**

| **Country** | **Study** | **Number of strains per study** | **Number of strains per country** |
| --- | --- | --- | --- |
| Belgium | This study | 2 | 2 |
| Czech Republic | Pepin-Puget et al. | 5 | 5 |
| France | Zamudio et al. | 34 | 42 |
|  | Pepin-Puget et al. | 2 |  |
|  | This study | 6 |  |
| Germany | Pepin-Puget et al. | 3 | 17 |
|  | This study | 14 |  |
| Hungary | This study | 3 | 3 |
| Italia | Pepin-Puget et al. | 4 | 13 |
|  | This study | 9 |  |
| Poland | Pepin-Puget et al. | 2 | 5 |
|  | This study | 3 |  |
| Spain | Pepin-Puget et al. | 6 | 9 |
|  | This study | 3 |  |
| Switzerland | Pepin-Puget et al. | 1 | 1 |
| The Netherlands | Pepin-Puget et al. | 1 | 2 |
|  | This study | 1 |  |
| The United Kingdom | Pepin-Puget et al. | 9 | 10 |
|  | This study | 1 |  |

# Table IV. Method used to select *E. coli* isolates from cats and dogs in the included studies.

| **Study** | **Method used to select bacterial isolates** | **Years of sampling** |
| --- | --- | --- |
| Zamudio et al. | - Resapath network. Strains were collected from sick animals in French peripheral veterinary clinics. Prior to identification of the ESC-R gene, the *E. coli* strains were cultured on non-selective media. | 2008 – 2016 |
| Pepin-Puget et al. | - *E. coli* strains were collected from sick dogs and cats as part of the CEESA Compath II program in 12 European countries: Belgium, Czech Republic, France, Germany, Hungary, Italy, Netherlands, Poland, Spain, Sweden, Switzerland, and the United Kingdom. - Strains were sampled by veterinarians. One sample was collected per animal. Dogs and cats had not been treated with antibiotics in the previous four weeks. Only one animal per household, breeder, or kennel was included. | 2013 – 2014 |
| This study | - *E. coli* strains were collected from sick dogs and cats as part of the CEESA Compath III program in 12 European countries: Belgium, Czech Republic, France, Germany, Hungary, Italy, the Netherlands, Poland, Spain, Sweden, Switzerland, and the United Kingdom. - Strains were sampled by veterinarians. One sample was collected per animal. Dogs and cats had not been treated with antibiotics in the previous four weeks. Only one animal per household, breeder or kennel was included. | 2017 – 2018 |

# Table V. Sequencing and assembly methods used for each study.

| **Study** | **Illumina device** | **Single or paired ends** | **Read size** | **Assembly method** |
| --- | --- | --- | --- | --- |
| Zamudio et al. | MiSeq | Pair ends | 2*301 pb | See material and methods section |
| Lipworth et al. | HiSeq/MiSeq | Pair ends | 2*100 or 2*250 pb | See material and methods section |
| Kallonen et al. | HiSeq 2000 | Pair ends | 2*100 pb | See material and methods section |
| Roer et al. | MiSeq | Pair ends | 2*250 pb | See material and methods section |
| Sepp et al. | HiSeq 2500 | Pair ends | 2*100 pb | FASTX-Tooklkit  Velvet v.1.2 |
| de Lastours et al. | NextSeq | Pair ends | 2*150 pb | Shovill v.1.0.4  SPAdes v3.13.1 |
| Pepin-Puget et al. | NextSeq | Pair ends | 2*150 pb | See material and methods section |
| This study | NextSeq | Pair ends | 2*150 pb | See material and methods section |


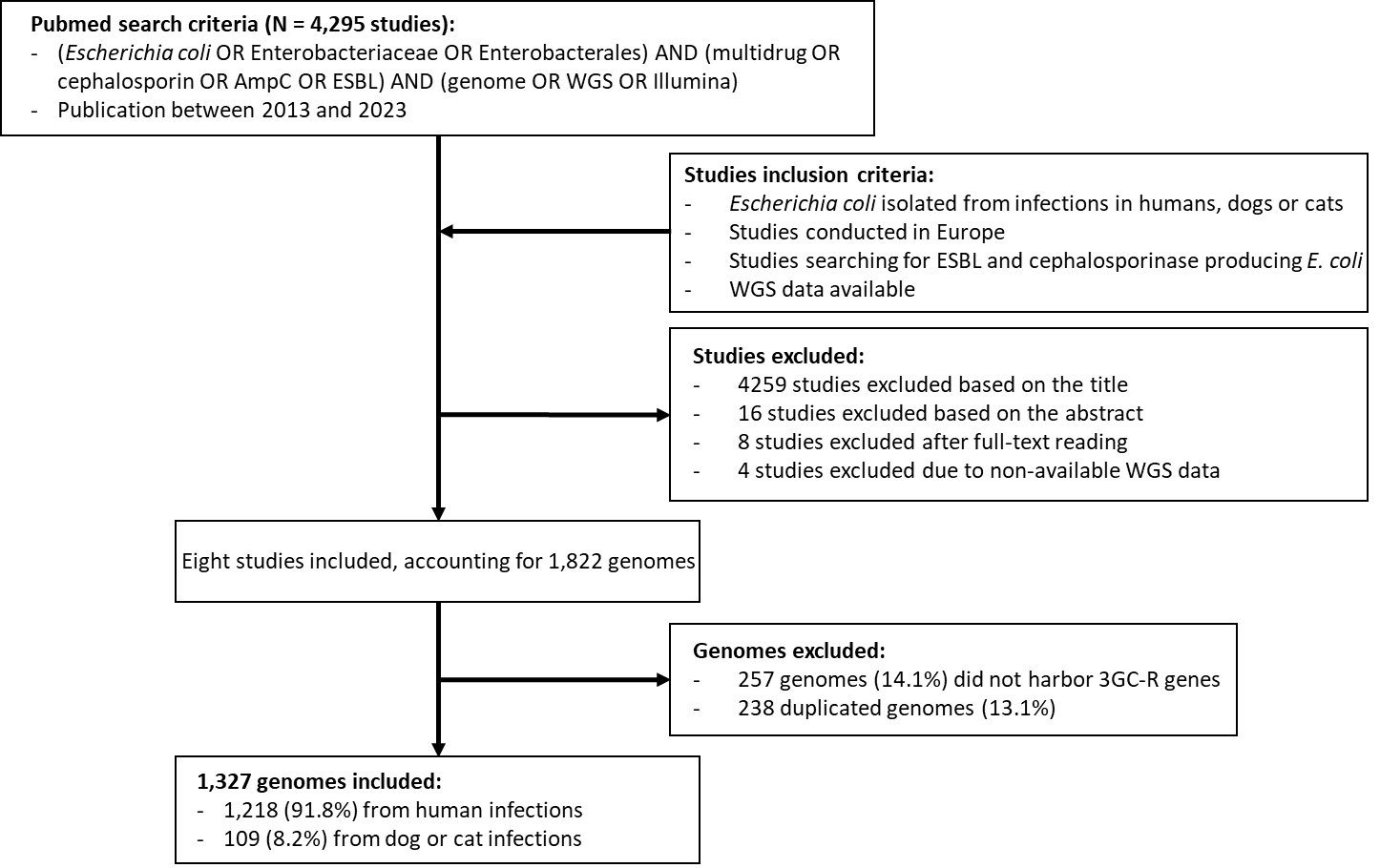


**Fig. 1. Flowchart of the studies and genomes included.**
